# Re-Positive SARS-CoV-2 With Respiratory Failure and Cerebrovascular Accident: Is This a Reinfection?

**DOI:** 10.7759/cureus.15825

**Published:** 2021-06-22

**Authors:** Shalanki Baiswar, Rea Mittal, Tarkeshwar Tiwary, Praveen Jinnur

**Affiliations:** 1 Internal Medicine, WellSpan Health, Chambersburg, USA; 2 Neurology, Penn State College of Medicine, Hershey, USA; 3 Pulmonology, WellSpan Chambersburg Hospital, Chambersbrug, USA; 4 Pulmonary/Sleep Medicine, Essentia Health, Fargo, USA

**Keywords:** clinical case report, stroke, covid-19 reinfection, covid reactivation, covid-2019, covid, covid-19

## Abstract

The coronavirus disease 2019 ( COVID-19) pandemic is a global pandemic where healthcare providers are concerned about the reinfection of recovered patients. The reinfection with COVID-19 is not common and considered less likely, but as time passes by, there are reports of patients becoming positive after having tested negative previously. Here, we report a case of a 28-year-old male with diabetes mellitus type 1, hypertension, and end-stage renal disease on hemodialysis who presented initially in April 2020 with nausea, vomiting, and dyspnea. His severe acute respiratory syndrome coronavirus 2 (SARS-CoV-2) polymerase chain reaction (PCR) came back positive. He left against medical advice but was followed as an outpatient in the dialysis unit where he continued with dialysis in isolation for positive COVID-19 as per the dialysis unit guidelines. He presented three months later with altered level of consciousness in the setting of diabetic ketoacidosis. He also had gastrointestinal bleed and cerebrovascular accident. There was a strong possibility of reinfection in this patient as he was tested negative after the initial infection and then tested positive three months later, presenting with a different set of symptoms and more severe disease on his second admission.

## Introduction

Coronavirus disease 2019 (COVID-19) was first discovered in December 2019 and was declared a global pandemic by the WHO in March 2020 [1]. Healthcare providers are concerned about the possibility of reinfection or reactivation in COVID-19 patients. There are currently few confirmed cases of reinfection with severe acute respiratory syndrome coronavirus 2 (SARS-CoV-2), with mixed evidence regarding the symptomatic intensity of reinfection, where most patients present mildly but some patients present more aggressively [2-5]. Here we discuss a case that raises the possibility of reinfection with a distinct clinical presentation.

## Case presentation

A 28-year-old male with diabetes mellitus type 1, hypertension, and end-stage renal disease on hemodialysis with multiple past admissions for diabetic ketoacidosis and uncontrolled hypertension presented to the emergency department on April 17, 2020, for nausea and vomiting. His COVID-19 test was positive on the same day. He was managed symptomatically; however, he left against medical advice from the hospital 48 hours later and was instructed to follow the isolation precautions. He continued hemodialysis as an outpatient with COVID-19 isolation and precautions as per the dialysis unit protocol. The patient was supposed to be tested for COVID-19 as per the dialysis unit protocol but did not get the test done. Finally, he got the test done on May 15, 2020, which was inconclusive. He was retested on May 21, 2020, when it came back positive. He was tested again on June 2, 2020, and was negative. The patient continued to have intermittent back pain, nausea, vomiting, cough, and shortness of breath, which were mostly related to non-adherence with dialysis and shortening the dialysis treatment time.

**Figure 1:**
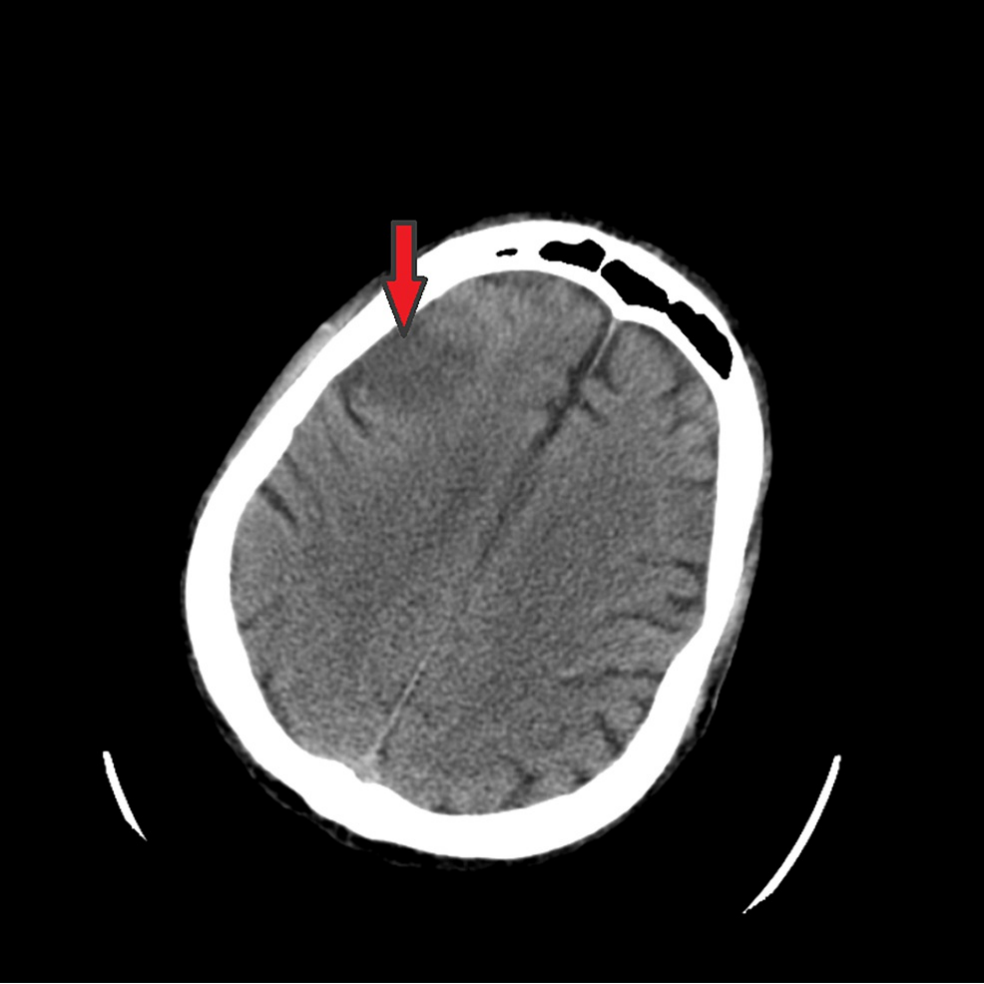
CT scan of the head demonstrating right frontal cytotoxic edema

The patient was again admitted on August 17, 2020, for headaches and altered mental status. His mother provided pertinent history due to his altered mental status. He had weeklong severe headaches and left-hand weakness. As per the documentation by the emergency department, the patient had altered mental status but gradually became somnolent. Based on the examination by different providers during the emergency department encounter, his motor and sensation abilities were initially grossly intact. The patient eventually became unresponsive and was intubated for airway protection. The neurology service was consulted, and as per their examination at this point in time, he was not opening his eyes to verbal or noxious stimuli. The patient mildly withdrew the right upper extremity to noxious stimuli, but no withdrawal was noted in the left upper extremity to noxious stimuli. Triple flexion was noted bilaterally in the lower extremities with and without noxious stimuli. His head CT scan showed mild right frontal cytotoxic edema consistent with acute or subacute infarct (Figure 1). CT angiogram of the head and neck did not show any stenosis or thromboembolic disease. Chest X-ray showed bilateral pleural effusion and pulmonary congestion. He was difficult to arouse and became tachypneic with respiratory failure for which he was emergently intubated and was started on mechanical ventilation. He was noticed to have upper gastrointestinal (GI) bleed and blood in the orogastric tube after intubation. Upper endoscopy was performed, which showed esophagitis, no active bleeding, minimal duodenitis, and gastritis. CT of the chest showed bilateral pleural effusion and consolidation of the right upper lobe and lingula suspicious for pneumonia. He was therefore diagnosed with pneumonia as well. Sputum cultures grew *Klebsiella oxytoca*, which was treated with IV antibiotics including piperacillin/tazobactam and ceftriaxone. The infectious disease service was consulted, and this was considered a chronic infection and therefore no further treatment for COVID-19 was recommended.

**Figure 2:**
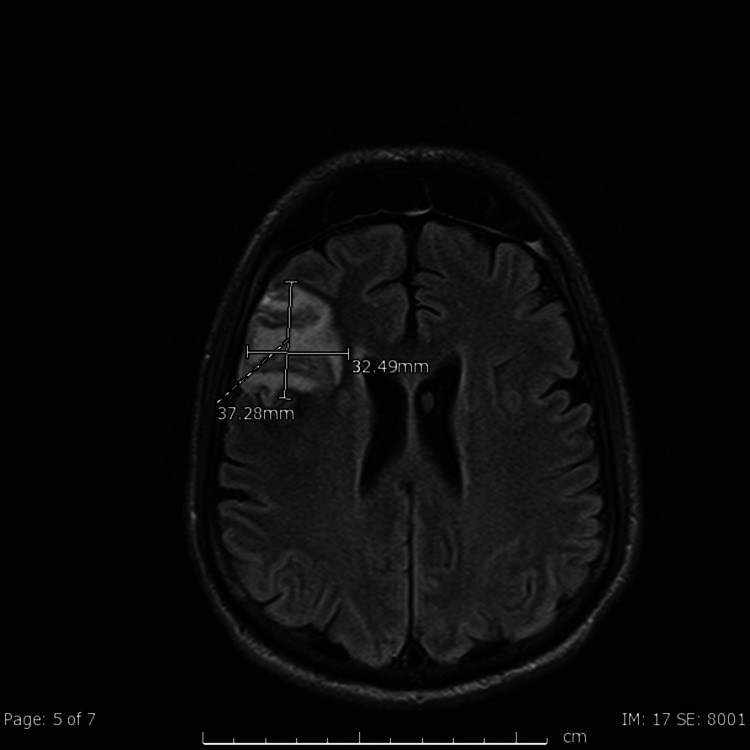
Axial FLAIR MRI of head demonstrating multifocal areas of acute infarct involving the right temporal lobe and right frontoparietal junction FLAIR, fluid-attenuated inversion recovery

MRI of the brain performed after the patient was stabilized showed multifocal areas of acute infarct involving predominantly the temporal lobes and right frontoparietal junction, with suspected right middle frontal gyrus subacute infarct (Figure 2). The patient was eventually extubated and transferred to the regular floor. Rehab unit was recommended but the patient refused it and was discharged home.

This patient and his clinical course most likely represent reinfection of COVID-19, as evidenced by a negative test followed by a positive test almost three months later. He presented with different symptoms on his admissions for COVID-19 on two separate occasions. His first presentation was for nausea, vomiting, and dyspnea. His next presentation three months later was for cerebrovascular accident (CVA). He had chronic/intermittent dyspnea at baseline, for which he required mechanical ventilation on his second admission for worsening encephalopathy and airway protection. He had an upper GI bleed, which could have been due to COVID-19 as well due to thromboembolic phenomenon. Reinfection is a strong possibility in this patient; however, it was not confirmed with viral cultures and genetic tests. The patient continues to have neurological deficits post-discharge, specifically left upper extremity weakness.

## Discussion

This case strongly raises the possibility of reinfection. There are some case reports that have shown positive PCR test after being negative in the past and the patients presented with similar symptoms on both occasions, unlike our patient, who presented with GI/respiratory symptoms on his first episode but GI symptoms/CVA on the second episode [6]. Although there can be alternative explanations for his GI bleed, CVA, and respiratory failure, all this can be explained by the syndrome of SARS-CoV-2. GI bleed and CVA could be due to the thromboembolic complications of COVID-19. He also had respiratory failure, which can be attributed to pneumonia and was treated with antibiotics, but pulmonary pathology secondary to COVID-19 cannot be ruled out.

COVID reinfection can occur with at least four other common human coronaviruses such as 229E, NL63, and OC3, all of which generally cause milder respiratory illness. Viral burden in initial COVID-19 infection typically peaks early in the illness and then declines as antibodies develop and antibodies titer rise over the next two to three weeks [7]. Serological assays to detect SARS-CoV-2 antibodies are available but might not be able to confirm the reinfection.

Immunosuppressive factors such as medications and comorbidities contribute to impaired viral clearance and favor SARS-CoV-2 reactivation, which should be considered in our patient due to his multiple comorbidities including type 1 diabetes mellitus and end-stage renal disease.

Currently, cases of reinfection with SARS-CoV-2 are rare; however, there is growing concern among healthcare providers about the risk of recurrence or reactivation in a clinically distinct and critical presentation [4-6]. Clinical suspicion of SARS-CoV-2 reinfection is warranted in patients who become positive after a negative test, including clinically distinct presentations. Future viral spread in these patients is also of concern, where patients may still be virus carriers, and the average contagious period of SARS-CoV-2 infected patients is 20 days [8-9]. More studies are needed to determine the reinfection or recurrence or reactivation of the SARS-CoV-2 virus in patients who have a positive test following a negative test [8].

## Conclusions

In conclusion, if a patient has a positive test after a negative test in the past, sampling error and prolonged viral shedding should be considered along with the possibility of reinfection. We do not clearly understand the immune response for SARS-CoV-2 at this time, and reinfection remains a strong possibility in our patient as his symptoms were consistent with two clinically distinct COVID-19 syndromes with almost complete resolution of his initially presenting symptoms and negative test in between the two episodes. Although we do not have the viral cultures to prove the reinfection, it is a significant concern in our patient and should be strongly considered.
